# Advances and Challenges in Pharmaceutical Therapies to Prevent and Repair Cochlear Injuries From Noise

**DOI:** 10.3389/fncel.2019.00285

**Published:** 2019-06-26

**Authors:** Eric C. Bielefeld, Megan J. Kobel

**Affiliations:** ^1^Department of Speech and Hearing Science, The Ohio State University, Columbus, OH, United States; ^2^Department of Otolaryngology-Head & Neck Surgery, The Ohio State University Wexner Medical Center, Columbus, OH, United States

**Keywords:** noise, cochlea, blood-labyrinth barrier, pharmaceutical, otoprotection, rescue

## Abstract

Noise induces a broad spectrum of pathological injuries to the cochlea, reflecting both mechanical damage to the delicate architecture of the structures of the organ of Corti and metabolic damage within the organ of Corti and lateral wall tissues. Unlike ototoxic medications, the blood-labyrinth barrier does not offer protection against noise injury. The blood-labyrinth barrier is a target of noise injury, and can be weakened as part of the metabolic pathologies in the cochlea. However, it also offers a potential for therapeutic intervention with oto-protective compounds. Because the blood-labyrinth barrier is weakened by noise, penetration of blood-borne oto-protective compounds could be higher. However, systemic dosing for cochlear protection from noise offers other significant challenges. An alternative option to systemic dosing is local administration to the cochlea through the round window membrane using a variety of drug delivery techniques. The review will discuss noise-induced cochlear pathology, including alterations to the blood-labyrinth barrier, and then transition into discussing approaches for delivery of oto-protective compounds to reduce cochlear injury from noise.

## Introduction

Noise-induced hearing loss is a highly prevalent condition due to the combination of high-level noise in the workplace and in different non-occupational settings. Many countries have imposed occupational noise standards to minimize the number of workers sustaining NIHL. However, these standards are predicated upon a significant period of recovery during non-work hours. As recreational or simply non-occupational noise sources increase, the recovery interval for those exposed to noise decreases. Thus, NIHL continues to be a significant health hazard for many societies. In many of those societies, acoustic protection devices are widely available, but the expense, diminished auditory input, and discomfort associated with wearing them reduces their use in several vulnerable populations. In addition, for the devices to be effective, they must be used properly, which can be challenging for some who are without access to specific training. Further, they are impractical in a number of settings where communication is needed, environmental noise perception is critical for safety, or where the noise exposures cannot be anticipated. Therefore, there has been an ongoing need to develop pharmaceutical approaches to reduce susceptibility to cochlear injury from noise.

Pharmaceutical protection from hearing loss has been explored against a number of the causes of acquired sensorineural hearing loss, including noise (see below), ototoxic medications (Campbell et al., 1996; Chen et al., 2007; Bielefeld et al., 2013), auto-immune disorders (Trune et al., 1999; Van Wijk et al., 2006), and aging (Bielefeld et al., 2008; Vlajkovic et al., 2011). These conditions have several common sites of pathology in the cochlea and share many of the same underlying mechanisms. All of the pharmaceutical approaches to preventing cochlear injury share the same challenges of the barriers of tissue uptake into the cochlea, and cellular uptake into the populations most vulnerable to injury. Despite the commonalities across these different cochlear insults, there are also many differences, and each carries its own set of challenges. For example, pharmaceutical protection from ototoxic drug-induced hearing loss has the advantage of a well-defined window for when cochlear injury might take place because the schedule of ototoxic drug delivered is clearly defined for the clinical patient. However, pharmaceutical protection is complicated because it must occur without comprising the health benefits the ototoxic drug offers. For auto-immune disorders, the onset is often sudden and without any warning. For that reason, pharmaceutical treatment is limited to rescue approaches. In rescue approaches, the treatment compound is given after the insult to minimize the amount of permanent injury. For age-related hearing loss, the challenge lies with the fact the hearing loss occurs gradually over a long period of time without a clearly identifiable underlying pathology that is consistent from patient to patient.

Noise presents a unique insult to the cochlea. However, addressing a pharmaceutical protection strategy for noise requires consideration of many of the challenges associated with other cochlear insults. Noise can induce simultaneous metabolic and mechanical changes that can injure the organ of Corti in both overlapping and separate ways (Henderson et al., 2006). The relative contribution of metabolic and mechanical damage to the cochlea is dictated by the sound pressure level, the duration, the frequency content, and the kurtosis factor of the noise exposure. Kurtosis factor estimates the deviation of a sample from the Gaussian distribution (Qiu et al., 2006) and can be used as a measure of the randomness of a noise exposure over time, with sensitivity to the peaks present in the exposure (Hamernik et al., 2003). Noise also offers challenges to pharmaceutical protection from the timing and dosing perspectives. Hazardous noise exposures can occur repeatedly over a period of months, years, or decades. In those cases, pharmaceutical protection from noise requires a compound with high efficacy that can be delivered to the cochlea without the systemic side effects associated with repeated dosing. Noise exposures can also occur suddenly without any opportunity to prepare. In those instances, there is a therapeutic window for rescue after the noise exposure in which the damage can be potentially mitigated pharmaceutically. This rescue approach carries an urgent need for the compound to reach the injured cells as quickly as possible in potentially high doses.

Further complicating the issue of drug delivery into the noise-exposed cochlea is the potential for noise exposure to alter blood flow to the cochlea, and to alter the BLB (discussed in more detail below). These alterations to the BLB may have the effect of increasing the penetration of rescue compounds into cochlear tissue, but also may change the distribution of the compounds within the different compartments and cells of the cochlea.

The current review will describe the myriad of strategies that have been tested with the goal of achieving pharmaceutical protection from noise injury, with a focus on the different approaches that have been taken to optimize drug delivery into the noise-exposed cochlea. Particular emphasis will be placed on the drug delivery challenges that are unique to noise protection. The review will begin with a description of the pathophysiologic changes that occur in the cochlea during and after exposure to hazardous noise. The impact of noise on the BLB will be discussed, along with implications that those changes may have on drug delivery. Finally, a review of different approaches that have been taken to treat the ear pharmaceutically through local drug delivery to the cochlea or systemic delivery in protection (drug delivery before noise exposure) and rescue (drug delivery after noise exposure) paradigms will be presented in order to critically evaluate the approaches that offer the most promise for clinical application in the noise-exposed patient population.

## Noise-Induced Injury to the Cochlea and Blood-Labyrinth Barrier

Noise exposure causes a broad set of physical changes in cochlear structures, including the organ of Corti, neuronal terminals, spiral ligament, and stria vascularis (Wang Y. et al., 2002; Henderson et al., 2006; Ohlemiller, 2008). Mechanical injuries in the organ of Corti can result in holes in the reticular lamina (Ahmad et al., 2003), damaged stereocilia (Slepecky et al., 1981), and disconnection of the hair cells from supporting cells (Henderson et al., 2006). Metabolic stress includes proliferation of reactive oxygen species in the cochlea (Yamane et al., 1995; Ohlemiller et al., 1999; Yamashita et al., 2004). OHC loss has frequently been linked to PTS, and OHCs are known to be vulnerable to acoustic-related injury (Bohne and Harding, 2000; Wang Y. et al., 2002; Chen and Fechter, 2003). However, there is a poor correlation between OHC loss and degree of PTS in many mammalian species (Ohlemiller et al., 2000; Chen, 2002; Chen and Fechter, 2003), and in those cases, IHC loss can be better correlated with PTS (Nordmann et al., 2000; Harding et al., 2002). IHC loss after noise exposure is considerably smaller than OHC loss and generally occurs after OHC loss (Nordmann et al., 2000; Harding et al., 2002).

In addition to hair cell damage and loss, noise exposure can also lead to damage to Type I afferent neurons and their peripheral processes, which may underlie TTS (Robertson, 1983; Puel et al., 1998; Pujol and Puel, 1999). Swelling and vacuolization of afferent terminals after noise exposure may be due to glutamate excitotoxicity (Puel et al., 1998). After hair cell loss due to noise exposure, SGNs can undergo secondary degeneration, especially in regions with destroyed IHCs (Stankovic et al., 2004; Sugawara et al., 2005). Long-term survival of neurons is enhanced by the presence of intact supporting cells (Stankovic et al., 2004; Sugawara et al., 2005). Supporting cells sit in close proximity to the unmyelinated portion of the SGNs near the hair cell synapses and express many markers similar to the glial cells in the central nervous system that provide necessary trophic support (Rio et al., 2002; Stankovic et al., 2004). In cochlear regions with IHC loss, degeneration of peripheral axons has been detected at 1 week after noise exposure, and degeneration of cell bodies at 8 weeks after noise exposure (Wang Y. et al., 2002).

Since hair cell loss occurs directly after noise exposure and SGN degeneration follows a much longer time course, this degeneration was believed to occur secondarily to hair cell loss (Stankovic et al., 2004; Kujawa and Liberman, 2009). However, evidence suggests that primary SGN loss can occur in the absence of hair cell loss over a period of several months to years after noise exposure (Kujawa and Liberman, 2006, 2009; Lin et al., 2011).

**FIGURE 1:**
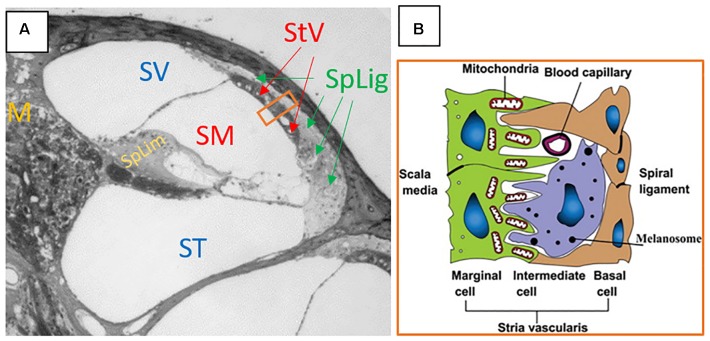
**(A)** Cross section of the cochlea. SV, scala vestibuli; SM, scala media; ST, scala tympani; StV, stria vascularis; SpLig, spiral ligament; M, modiolus; SpLim, spiral limbus. Note how spiral ligament surrounds stria vascularis, as it represents a connection point between substances in the periliymph of scala vestibuli and scala tympani, with the endolymph of scala media through the stria vascularis. The orange rectangle is a cross section of stria vascularis and spiral ligament that is schematized in panel **(B)**. **(B)** The schematic structure of the stria vascularis. Please note the capillary running through the middle of the stria vascularis between the basal and marginal cells and above the intermediate cell. Tight junctions, the basement membrane, pericytes, and perivascular resident macrophage type-melanocytes create the BLB separating the blood from the intra-strial space. Panel **(A)** is an image kindly supplied by Dr. Jianxin Bao of Northeast Ohio Medical University. Panel **(B)** is a figure reproduced from Jin et al. (2009), Cochlear homeostasis and its role in genetic deafness – Scientific Figure on ResearchGate. Available from: https://www.researchgate.net/figure/The-schematic-structure-of-the-stria-vascularis_fig1_275552111. Creative Commons License CC BY-NC-ND 4.0.

Protection of the cochlea and vestibular end organs from invasion by external pathogens comes in part from the BLB. The cochlea is characterized by the presence of three distinct fluid compartments, two of which are filled with perilymph and one with endolymph. Perilymph is characterized by its 0 mV electrical charge and ionic composition consistent with blood plasma or CSF. Scala media also contains cortilymph in the space underneath the reticular lamina, but above the basilar membrane. This fluid is ionically the same as perilymph, and bathes the cells of the organ of Corti below their apical surfaces. See Figure 1A for a representative cross section of the cochlea showing the three chambers. The exact origins of perilymph and cortilymph from within the body are unknown, as it may be filtered from plasma or be CSF that has entered the labyrinth through the cochlear aqueduct (Kaupp and Giebel, 1980) or via the internal auditory meatus (Glueckert et al., 2018). Endolymph is the fluid of scala media above the reticular lamina that drives the depolarization of the hair cells through its positive endocochlear electrical potential and its high K+ concentration. The endocochlear potential is generated by the active mechanism in the marginal cells of stria vascularis. Therefore, in order to restrict access to cells of the organ of Corti, the BLB must restrict access from the bloodstream into both the endolymph, via stria vascularis, and the perilymph/cortilymph. The intra-strial barrier is characterized by many of the same mechanisms that define the blood-brain barrier. Materials must cross from the bloodstream, through the capillaries within stria vascularis, and those capillary walls represent a major site of the BLB. See Figure 1B for a schematic of a cross section of the stria vascularis and the capillaries that run through it (Jin et al., 2009). The endothelial cells on the walls of the strial capillaries are connected by tight junctions (Juhn, 1988). Those endothelial cells are supported by a basement membrane and population of pericyte cells (Shi, 2009), the latter of which may have a regulatory role in cochlear blood flow (Dai et al., 2009). Further, the BLB at the level of the strial capillaries is formed in part by processes from perivascular resident macrophage type-melanocyte cells to further seal the capillary walls (Shi, 2010; Zhang et al., 2012). See Shi (2016) for a detailed review of the structure of the strial capillary BLB. The pericytes and perivascular resident macrophage type-melanocytes help to control the expression of the tight junctions between the endothelial cells (Neng et al., 2013). The blood-perilymph barrier is more difficult to characterize, due to the multiple possible origin points for the perilymph. If perilymph is derived from CSF through the cochlear aqueduct, then the blood-CSF barrier at the level of choroid plexi is separating the perilymph from the blood. In the choroid plexi, the blood-CSF barrier is formed by tight junctions between epithelial cells (Dando et al., 2014). Tight junctions of the epithelial cells of the arachnoid mater aid the blood-CSF barrier (Dando et al., 2014), as do tight junction of endothelial cells in the venous system within the sub-arachnoid space (Abbott, 2013). Locally at the level of the cochlea itself, tight junctions have been detected between the endothelial cells of capillaries in the modiolus (“M” on Figure 1A) and the mesothelial cells of the capillaries of spiral limbus (“SpLim” on Figure 1A; Jahnke, 1980), indicating a direct blood-perilymph barrier in the cochlea. Overall, due to the many possible points of entry from the blood to the perilymph, it is difficult to assess the blood-perilymph barrier anatomically. However, there has been research on noise effects on the transport of mannitol from the blood into the perilymph, and the finding was that impulse noise exposure does not alter the blood-perilymph barrier (Laurell et al., 2008). In contrast, there is evidence that noise exposure can damage the BLB at the level of stria vascularis. In stria vascularis, the pericyte cells’ connections to the capillary endothelial cells are weakened by high-level noise exposure (Shi, 2009). Horseradish peroxidase was found to leak from chinchillas’ strial blood vessels into the intra-strial spaces when the horseradish peroxidase was injected 25–30 min after a 22-min noise exposure at 122 dB SPL (Hukee and Duvall, 1985). Further, polyethyleneimine transport from strial blood vessels into cochlear tissues, specifically Reissner’s membrane, was increased in the ears of guinea pigs that had been exposed to a 105-dB SPL noise for 30 min (Suzuki et al., 2002). Such increases were not detected in spiral ligament, spiral limbus, or the basilar membrane. Increased permeability for lanthanum nitrate particles from cochlear blood vessels into strial tissue after 115 dB SPL noise for 4 or 6 h/day for 2 days was detected in albino guinea pigs (Wu et al., 2014). The noise-induced increase in permeability of the BLB has been attributed to noise-induced depletion of ATP causing a reduction in activity of the Na^+^-K-^+^-ATPase pump, which in turn, increases permeability of the tight junctions that characterize the functionality of the BLB (Yang et al., 2011).

Overall, the literature indicates that noise can cause a breakdown of the BLB at the level of the strial vasculature (Hukee and Duvall, 1985; Suzuki et al., 2002; Wu et al., 2014). The exact timing of those changes and recovery of the BLB after noise exposure are not explicitly clear, nor are the different dynamics of changes to the BLB with different noise parameters (level, duration, frequency content, and kurtosis). The reduction in the BLB seen after noise exposures indicates that, at least for a window of time after noise exposure, the cochlea may be maximally accessible for drug delivery. In the cases of oto-protective compounds, this could offer a window for optimal delivery of compounds in rescue paradigms.

## Rescue Approaches Using Systemic Drug Delivery

Systemic drug delivery methods are those delivered to the entire body, with the goal of getting a portion of the compound into the tissue that is in need. Systemic approaches offer the advantages of ease of drug delivery and the potential for repeated dosing to enhance therapeutic benefit. The disadvantages include exposure of other tissue systems in the body to compounds that could affect them negatively (side effects), potentially lower drug penetration into tissues that are sequestered from the blood, such as the brain or labyrinth, and uncertainty about pharmacokinetics of the compounds when delivered through different approaches. Because of the compromised BLB seen after noise exposure, drug access to cochlear fluids may be higher during a window of time after the noise has ended. That window of cochlear access may contribute to the effectiveness of rescue approaches to reduce NIHL. In pharmaceutical rescue from noise, i.p. and s.c. injections, have been tested extensively. A rescue approach to noise oto-protection is defined as one in which the pharmaceutical is delivered exclusively after the completion of the noise, and can be contrasted with a protection paradigm in which the oto-protective compound is delivered before the noise. Many protection paradigms also include dosing after the noise has finished, but the key distinction between protection and rescue is when the first dose of the oto-protective compound is delivered. For protection, that first dose is before the noise, and for rescue, the first dose (and all subsequent doses) is delivered after the noise. It should be noted that a very large number of additional pharmaceuticals have demonstrated oto-protective capabilities in protection paradigms, but the focus here is on rescue experiments in which the compounds are first delivered after the noise has ended. Therefore, we are excluding protection experiments that used systemic dosing that began before the noise exposure, even if the dosing continued after the noise exposure finished. For the following experiments reviewed, the experimental conditions are broadly summarized in Table 1 for comparisons.

**Table 1:** The experiments and noise exposure, drug, and animal model variables listed for each systemic rescue experiment discussed in Section “Rescue Approaches Using Systemic Drug Delivery.”

Experiment	Compound	Animal	Route	Noise duration and dB SPL	Time of first injection after noise	Subsequent injections	Efficacy of rescue
Wang Y. et al., 2002	Riluzole	Guinea pig	i.p.	30 min – 120 dB SPL	30 min, 1, 3, 6, 12, or 24 h	Once per day for 5 days	Partial
Tabuchi et al., 2006	MP	ddY mouse	unknown	4 h – 128 dB SPL	Immediate or 3 h	None	Partial
Bao et al., 2013	MP	C57BL/6J mouse	i.p.	30 min–110 dB SPL	24 h	None	Partial
Han et al., 2015	Dexameth-asone	C57BL/6J mouse	i.p.	1 h – 110 dB SPL	1 day	Once on day 4	Nearly complete
Zhou et al., 2009	MP	Guinea pig	i.m.	60 impulses – 165 dB pSPL	1 h	3 injections within 48 h	Partial
Campbell et al., 2007	D-methionine	Chinchilla	i.p.	6 h – 105 dB SPL	1 h	5 injections, one every 12 h	Nearly complete
Campbell et al., 2011	D-methionine	Chinchilla	i.p.	6 h – 105 dB SPL	3, 5, or 7 h	5 injections, one every 12 h	Nearly complete
Lo et al., 2013	D-methionine	Albino guinea pig	i.p.	6 h – 105 dB SPL	1 h	5 injections, one every 12 h	Partial
Coleman et al., 2007a	NAC or acetyl carnitine	Chinchilla	i.p.	6 h – 105 dB SPL	1, 4, or 12 h	5 injections, one every 12 h	Partial
Coleman et al., 2007b	AM-111	Chinchilla	i.p.	150 impulses – 155 dB pSPL	1 or 4 h	None	Partial
Bielefeld et al., 2011	KX1-004 or KX1-004+NAC	Chinchilla	s.c.	1 h – 112 dB SPL	1 and 24 h	None	Partial
Park et al., 2014	Renexin	C57BL/6 mice	Intra-gastric	1 h – 110 dB SPL	24 h	Once per day for 7 days	Nearly complete
Yamashita et al., 2005	Salicylate and trolox	Guinea pig	s.c. and i.p	5 h – 120 dB SPL	1 h, 1, 3, or 5 days	Twice daily until day 14	Partial
Vlajkovic et al., 2014	Adenosine amine congener	Wistar rat	i.p.	2 h – 110 dB SPL	12 h, 1, 2, or 3 days	Once every 24 h for 5 days	Partial

Riluzole, a wide-spectrum neuroprotective agent that can inhibit apoptosis and necrosis, was found to promote recovery from compound action potential threshold shift when given by i.p. injection daily for 5 days after a 30-min noise exposure (Wang J. et al., 2002). Injections beginning at 30 min and 1 h after the noise promoted recovery that was equivalent to when the drug was injected 30 min before the noise. When the first injection was at 3 h, the effectiveness diminished, and diminished further when the injections started at six or 12 h. By 24 h, the rescue effect was gone. This indicated a maximum window for the onset of therapeutic intervention between 12 and 24 h post-noise.

Glucocorticoids have been used in several different paradigms as an oto-protectant against NIHL (see below for local delivery approaches). Systemic rescue with MP was achieved, with partial, but statistically significant, reduction in PTS when MP was delivered immediately after noise to ddY mice. When given 3 h after the noise, there was no rescue effect on PTS. It should be noted that method of systemic delivery was not reported (Tabuchi et al., 2006). In contrast to the evidence that the window of rescue with MP is closed within 3 h of the noise, MP delivered to C57BL/6J mice via i.p. injection was able to reduce PTS when given at 24 h post-noise (Bao et al., 2013). Similarly, dexamethasone delivered by i.p.in C57BL/6J mice was able to provide nearly complete protection, measured 7 days after noise, when the dexamethasone was delivered one and 4 days after noise (Han et al., 2015). MP has also been used to induce rescue from impulse noise injury. Guinea pigs exposed to 165 dB pSPL impulses showed lower threshold shifts at 4 weeks when rescue treated with intra-muscular MP beginning at 1 h after the noise (Zhou et al., 2009).

D-methionine has been used to reduce oxidative stress-mediated cochlear injury from noise (Kopke et al., 2002) and ototoxic compounds (Campbell et al., 2007). As a rescue agent, it was delivered to chinchillas via i.p. injection starting 1 h after a 6-h noise exposure, with another four injections, one every 12 h, thereafter. The D-methionine injections reduced PTS across frequencies compared to controls injected with saline vehicle (Campbell et al., 2007). The rescue effect was also detected, without significant weakening, when the injections started at 3, 5, or 7 h after the same noise exposure (Campbell et al., 2011). A dose-dependent effect was also produced in the albino guinea pig, exposed to a 6-h noise and given i.p. injections D-methionine starting 1 h post-noise, and repeated every 12 h (Lo et al., 2013). The same experimental noise paradigm used by Campbell et al. (2007, 2011), injection pattern (every 12 h after initial dose), and animal model was also used to test the rescue effect of NAC and acetyl carnitine. Significant PTS reduction was found when the injections began 1 or 4 h after the noise concluded, but the effect was weaker when the injections began at 4 h compared to one. When the injections began at 12 h, no protection was found (Coleman et al., 2007a). AM-111, a proprietary compound containing D-JNK-1, a JNK inhibitor targeted to prevent noise-induced apoptosis, was also tested in chinchillas via i.p. injection 1 or 4 h after a noise exposure of repeated 155 dB pSPL impulses. Both injection times yielded significantly reduced PTS, with a slightly weaker effect from the 4-h injections (Coleman et al., 2007b). KX1-004, a Src-protein tyrosine kinase inhibitor, was also able to reduce PTS in chinchillas when given by s.c. injection one and 24 h after a 1-h noise exposure, either alone or in combination with NAC given i.p. (Bielefeld et al., 2011). Unpublished data from the authors of Bielefeld et al. (2011) using a rescue approach also indicated that additional injections after 24 h were not effective in creating a stronger rescue effect. These sets of findings corroborated the general notion that rescue intervention within a 12-h window after noise could significantly reduce PTS.

Despite the finding with riluzole that the critical window closes between 12 and 24 h after exposure, several studies have found rescue effects for interventions that commenced after 12 h post-noise. Renexin, a proprietary compound whose effects in the cochlea are believed to include increased blood flow and suppression of superoxide, improved recovery of thresholds by ∼5–10 dB in mice when given after a 1-h noise exposure (Park et al., 2014). The dosing was delivered intragastrically, beginning at 24 h post-exposure. Salicylate acts as a free radical scavenger, and has been shown to reduce gentamicin ototoxicity (Chen et al., 2007). Salicylate was also given s.c. in combination with i.p. trolox, an analog of vitamin E, as rescue agents against NIHL. The series of injections began at different times, 1 h, 1, 3, or 5 days after the 5-h noise exposure concluded. The injections were delivered twice daily until an endpoint of 14 days post-noise. Each of the injection series significantly attenuated PTS, OHC loss, and oxidative stress from the noise. However, the protective effect got progressively weaker with each delay in the onset of the injections, with the strongest protection from the injections starting at 1 h, and the weakest protection when the injections started at 5 days (Yamashita et al., 2005). Rescue after a 2-h noise exposure in rats with an adenosine amine congener, delivered i.p., reduced PTS when injections commenced 12 or 24 h after the noise, and the effect was equally strong with either starting time. There was a small protective effect when the injections began at 48 h, but no effect when started at 72 h (Vlajkovic et al., 2014).

Collectively, the experiments on rescue after noise exposure indicate that the optimal intervention time is essentially as soon as possible after the noise, and that the effect is weaker when the intervention begins after 24 h. However, they also indicate that intervention at or after 24 h can produce some rescue effect in some models. What remains uncertain is how much of a role the type of compound used, the animal model, the route of administration, the dose levels, the number and type of subsequent injections, and the acoustic parameters of the noise exposure have on the rescue effect. Finally, though the BLB is compromised after noise exposure, it is unknown whether increased permeability of the BLB affected any of the rescue efficacy of the compounds described. Drug penetration into the cells vulnerable to injury from noise is inferred by the efficacy of the compounds in conferring rescue. Pharmacokinetics studies after systemic dosing are needed to further understand how/when the drugs are reaching the cochlear cells, to assess the effects of noise-induced changes to the BLB, and to determine how to optimize drug penetration to the cells in need.

## Local Delivery of Oto-Protective Compounds to the Cochlea

**FIGURE 2:**
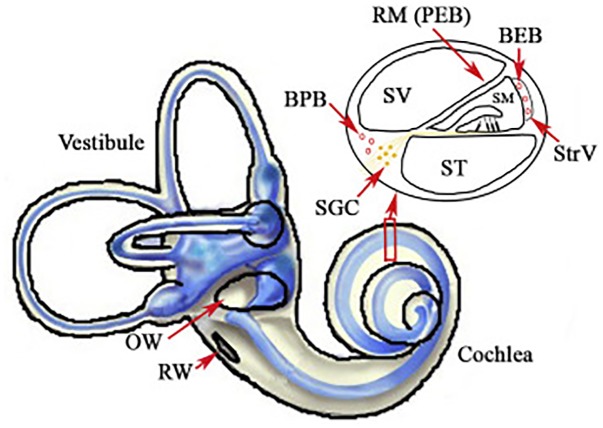
Illustration of the inner ear, with highlights of the oval (OW) and round windows (RW). The inset displays a cross section of the cochlear chambers. Scala vestibuli (SV) and scala tympani (ST) are filled with perilymph and are accessible through local delivery of compounds into the middle ear space or through a cochleostomy. Scala media (SM) is filled with endolymph and separated from the blood by the blood-strial barrier in stria vascularis (StrV). Figure reproduced from Zou et al. (2016). Available from: https://www.sciencedirect.com/science/article/pii/S167229301630068X. Creative Commons License CC BY-NC-ND 4.0.

For protection or rescue from noise injury, a prime target has been local delivery of compounds directly into the cochlea. This approach avoids major concerns about systemic side effects. Access to the cochlea through diffuse through the RWM and/or the oval window membrane provide direct access to the perilymph of scala tympani and scala vestibule directly, as does a cochleostomy [see Figure 2 from Zou et al. (2016) for schematic representation of access to the perilymph through the oval window and RWM]. This would provide access to the cells of the organ of Corti via diffusion from the cortilymph (see blue arrow on the inset cross section diagram of Figure 2). Scala media and the endolymph are much harder to access, and therefore local delivery does not necessarily circumvent the BLB at the level of stria vascularis (Figure 1). Thus, compounds that are dependent upon crossing from the endolymph through the transduction channels of the stereocilia in order to penetrate the hair cells may not be effective in local delivery. Further, there are challenges with sustaining the compound within the cochlear tissues and optimizing drug delivery in the least invasive manner possible. Drug delivery to the cochlea through local delivery via the RWM has received significant attention due to increases in perilymph concentration (Salt and Plontke, 2009; Plontke and Salt, 2018) and reduction in plasma serum levels (Parnes et al., 1999; Chandrasekhar et al., 2000; Chen et al., 2003a; Bird et al., 2007; Salt and Plontke, 2009). However, applicability of RWM application clinically is restricted to agents that are efficacious when administered after trauma, as patients are unlikely to elect to undergo an invasive procedure prior to known exposure. The RWM acts as a semi-permeable membrane and application can be accomplished via IT injection into the middle ear space or, for more precise and sustained deliveries, using hydrogel formulations (Paulson et al., 2008; Borden et al., 2011) or osmotic mini-pump perfusion (Salt and Plontke, 2005). The assumed route of drug delivery is diffusion across the RMW with secondary diffusion through the oval window (Bachmann et al., 2001; Salt et al., 2012a; Salt and Hirose, 2018) and possible entrance via the otic capsule in some animal models (Mikulec et al., 2009). The bony otic capsule in the apical turns is very thin in some species, such as guinea pigs and chinchillas. Drugs applied filling the entire bulla rather than irrigating against the RWM, showed significantly higher apical concentrations suggesting permeability of the bony otic capsule (Mikulec et al., 2009). Drugs applied via IT injections can be lost in the middle ear via the Eustachian tube, middle ear vasculature and lymphatics (Salt and Plontke, 2018), and the use of gel formulations has been shown to stabilize retention in the middle ear (Wang et al., 2009, 2011). Protective effects from noise exposure are achieved through actions on multiple pathways underlying NIHL, including anti-inflammatory (Chi et al., 2011), anti-apoptotic (Wang et al., 2007) and antioxidant and free radical scavenging (Alagic et al., 2011). Overall protective effects for local application are typically greater than systemic delivery methods (Wang J. et al., 2002; Han et al., 2015) due to the presumed higher level of detectable concentrations in the cochlea. Similarly, prolonged applications rather than single applications have been seen to be more efficacious (Harrop-Jones et al., 2016) due to the presumed increase in duration of delivery.

Perilymphatic fluid is the primary medium for local pharmacotherapeutic administration. Inner ear fluids do not show appreciable movement or “stirring” and locally applied drugs are dependent on passive diffusion rates (Salt and Ma, 2001; Salt, 2002; Salt and Plontke, 2005). Dependent on perilymph sampling methods, local administration results in substantial base-to-apex gradients in agent concentration (Laurell et al., 2002; Mynatt et al., 2006; Bird et al., 2007; Plontke et al., 2007; Salt, 2008; Creber et al., 2018), as reflected in cochlear tissue labeling (Laurell et al., 2002; Grewal et al., 2013; Creber et al., 2018). These gradients are lower for hydrogel applications (Salt et al., 2011) or intracochlear injections in comparison to IT solution injections into the middle ear space (Hahn et al., 2012). Additionally, a significant variability in perilymph concentrations is seen between individual humans or animal species for RWM local delivery (Bird et al., 2007) which is diminished for hydrogel (Borden et al., 2011; Salt et al., 2011) and intra-cochlear delivery (Hahn et al., 2012).

Glucocorticoids, including MP and dexamethasone, have received significant research in inner ear pharmacokinetics and tissue uptake, more so than for other agents, as local administration is used clinically for treatment of several inner ear disorders including NIHL (Bird et al., 2007; Swan et al., 2008; Zhou et al., 2009; Stachler et al., 2012; Trune and Canlon, 2012). Glucocorticoids are effective for protection from hazardous impact of noise through suppression of cochlear responses to oxidative stress, ischemia, and inflammation. Glucocorticoids have been shown to modulate the inflammatory response through inhibition of tumor necrosis factor α–induced cytokine secretion from cochlear spiral ligament fibrocytes *in vitro*, increasing cochlear blood flow *in vivo*, and enhancing glutathione biosynthesis in the cochlear spiral ganglion (Shirwany et al., 1998; Maeda et al., 2005; Nagashima and Ogita, 2006). While several have investigated the impact of RWM drug administration on noise exposure and many studies have examined the pharmacokinetics of glucocorticoids in the inner ear, there have been relatively few studies examining both in the same population.

In general, local administration of MP or dexamethasone leads to greater perilymph concentration than systemic application (Bird et al., 2007; Yang et al., 2008; Grewal et al., 2013; Creber et al., 2018; Lee et al., 2018). For example, in humans, local IT administration of 40 mg of MP led to a 126-fold greater perilymph concentration after a single 1 mg/kg IV injection and 33-fold increase after 10 mg/kg IV infusion (Bird et al., 2007). After RWM application of 20% dexamethasone in guinea pigs, a higher tissue uptake and greater perilymph concentration of approximately two orders of magnitude than that after systemic IV delivery of 2 mg/kg (Creber et al., 2018). Additionally, higher and more prolonged intracochlear uptake was seen after administration of 3 IT injections of 20 μL of 10 mg/mL of fluorescein isothiocyanate-labeled dexamethasone in comparison to 3 i.p. injections of 60 μG/100 g (Lee et al., 2018). Tissue labeling after local administration mirrors glucocorticoid receptor distribution with the highest labeling in the SGNs, organ of Corti, and lateral walls (Lee et al., 2018) corresponding to the highest concentration of receptors in cochlear structures (Zuo et al., 1995). Dexamethasone perilymph elimination half-life has been calculated to be 111 min (Salt et al., 2012b) with peak glucocorticoid receptor activation, dexamethasone tissue labeling, and perilymph concentration 60 min after local application (Hargunani et al., 2006; Yang et al., 2008; Grewal et al., 2013). After this, perilymph concentrations after IT injection show elimination from perilymph by around 6 h (Parnes et al., 1999; Bird et al., 2007; Yang et al., 2008). Dexamethasone labeling in cochlear tissue is present up to 7 days after administration (Lee et al., 2018), but significant decreases in uptake and labeling are seen by 12 h (Wang et al., 2011; Grewal et al., 2013). Greater dexamethasone uptake and immunofluorescent labeling has been reported in IHCs compared to OHCs in mice (Grewal et al., 2013), but this selective uptake has not been seen across species (Creber et al., 2018). Cochlear tissue penetration exhibited a base-to-apex gradient, similar to perilymph concentrations, suggesting that tissue penetration is proportionally related to perilymph concentrations (Grewal et al., 2013; Creber et al., 2018). However, this gradient was not seen in receptor activation in SGNs in guinea pigs (Creber et al., 2018), which indicates a dissociation between end organ target receptor activation and perilymph concentrations.

Local glucocorticoid administration prior to noise exposure has been shown to protect against compound threshold shifts measured 7 days after exposure (Takemura et al., 2004; Harrop-Jones et al., 2016). For the following experiments reviewed, the broad experimental parameters and protective effects are provided in Table 2 to allow comparison. Efficacy of pre-exposure IT injections are time-dependent with reduced protection seen by 24 h prior suggesting that early administration is limited due to clearance from the perilymph at the time of injury (Yildirim et al., 2005; Harrop-Jones et al., 2016). Sustained release using thermoreversible hydrogels as a drug carrier has been shown to increase dexamethasone concentrations and provide lasting exposure in guinea pigs and sheep for days to weeks (Wang et al., 2009, 2011; Borden et al., 2011). However, protective effects have been limited (Yildirim et al., 2005; Harrop-Jones et al., 2016; Mamelle et al., 2018; Zhu et al., 2018). Zhu et al. (2018) utilized hydrogels containing 6% dexamethasone or 30% TAAC prior to a 3-h 120 dB SPL noise exposure in guinea pigs. No PTS protection was achieved 28 days after exposure, but a small protective effect was noted at 7 days. As well, there was significantly higher preserved SGN density in the first turn in the dexamethasone group, suggesting possible protection against supra-threshold ABR amplitude loss (hidden hearing loss). No protection in thresholds or cochlear structures was noted for TAAC (Zhu et al., 2018). A similar lack of threshold protection was seen with 1% dexamethasone hydrogel application 48 h after a 1-h 100 dB SPL noise exposure in guinea pigs (Mamelle et al., 2018). However, use of 2 or 6% OTO-104, a poloxamer hydrogel containing dexamethasone, 1 day prior and up to 3 days after a 2-h 105 dB SPL noise exposure yielded significant functional protection, as assessed through ABR thresholds 7 days after exposure. No protective effect was noted at any time point utilizing IT injections of dexamethasone sodium phosphate solution (Harrop-Jones et al., 2016). The differing results between these studied utilizing hydrogels may be secondary to differences in drug formulation and noise exposure parameters which induced a larger threshold shift in the Zhu et al. (2018) study. Both Zhu et al. (2018) and Harrop-Jones et al. (2016) noted some protective effect at 7 days after exposure. However, the final time point in Zhu et al. (2018) was 28 days and animals were not followed to this time point by Harrop-Jones et al. (2016). Additionally, while a significant rescue effect was noted by Harrop-Jones et al. (2016) this was not seen by Mamelle et al. (2018) which may be attributed to differences in drug formulation or dosages (1% vs. 2% or 6%).

**Table 2:** The experiments and noise exposure, drug, and animal model variables listed for each systemic rescue experiment discussed in Section “Local delivery of Oto-Protective Compounds to the Cochlea.”

Experiment	Compound	Animal	Compound delivery	Noise duration and dB SPL	Time of first injection	Subsequent Injections	Efficacy
Zhu et al., 2018	Dexamethasone or TAAC	Guinea pig	Hydrogel	3 h – 120 dB SPL	Immediately post-noise	None	None (TAAC), Partial (DEX)
Mamelle et al., 2018	Dexamethasone	Guinea pig	Hydrogel	1 h – 100 dB SPL	48 h post-noise	None	None
Harrop-Jones et al., 2016	OTO-104 or Dexamethasone	Guinea pig	Hydrogel (OTO-104), solution (DEX)	2 h – 105 or 110 dB SPL	1 day prior, 2, 3, or 4 days post-noise	None	Partial (OTO-104), None (DEX)
Chi et al., 2011	Dexamethasone	Albino guinea pig	Gelfoam	80 impulses – 167 dB pSPL	1 day post-noise	None	Partial
Sendowski et al., 2006	MP	Guinea pig	Intracochlear osmotic pump	3 impulses – 170 dB pSPL	Immediately post-noise	Continuously for 7 days	Partial
Zhou et al., 2009	MP	Guinea pig	Solution	60 impulses – 165 dB pSPL	1 h	4 injections, one every 48 h	Partial
Han et al., 2015	Dexamethasone	C57BL.6J mice	Solution	1 h – 110 dB SPL	1 day post-noise	4 days after noise	Nearly complete
Alagic et al., 2011	D-methionine	Guinea pig	Solution	45 min – 100 dB	90 min prior	None	Partial
Zou et al., 2003	NAC	Guinea pig	Gelfoam	15 min vibration – 108 dB	24, 48, or 72 h or 7 days prior	None	None
Bielefeld et al., 2013	4-[2-amino-ethyl]benzenesulfonyl fluoride	Chinchilla	Solution	6 h – 106 dB SPL or 75 impulses – 155 dB pSPL	Immediately prior	None	Partial (impulse), none (continuous noise)
Wang et al., 2007	D-JNK-1	Guinea pig	Osmotic pump onto RWM or hydrogel	15 min – 130 dB SPL	30 min before, 1, 4, 6, 12 or 24 h post	Continuously for 7 days (osmotic pump), None (hydrogel)	Nearly complete if within 12 h
Wang et al., 2003	D-JNK-1	Guinea pig	Intracochlear osmotic pump	30 min – 120 dB SPL	2 days prior	Continuously for 7 days	Nearly complete
Coleman et al., 2007b	AM-111	Chinchilla	Hyrdogel or osmotic pump	150 impulses – 155 dB pSPL	1 or 4 h	None	Partial
Wang J. et al., 2002	Riluzole	Guinea pig	Intracochlear osmotic pump	30 min – 120 dB SPL	2 days prior	Continuously for 7 days	Partial
Chen et al., 2003b	Caroverine	Guinea pig	Gelfoam	1 h – 110 dB SPL	1 or 24 h post	None	Partial if 1 h, None if 24 h
Chen et al., 2004	Caroverine	Guinea pig	Gelfoam	1 h – 110 dB SPL	10 min prior	None	Partial

Rescue paradigms with delivery through an osmotic pump, or gelatin sponge, or IT injections has mixed effects on PTS protection despite reduction in HC loss (Sendowski et al., 2006; Zhou et al., 2009; Chi et al., 2011; Han et al., 2015). Chi et al. (2011) applied dexamethasone to the round window niche in guinea pigs using gelfoam 1 day after exposure to repeated 167 dB pSPL impulses. After application, perilymph sampling revealed a peak concentration at 15 min (over 5000 ug/ml) with a 17-fold decrease in concentration by 6 h (∼300 ug/ml). Significant reductions in threshold shifts and HC loss were noted 3 weeks post-exposure without accompanying protection of SGN morphology (Chi et al., 2011). In contrast, intracochlear MP for 7 days initiated immediately after exposure to 170 dB pSPL impulses in guinea pigs showed functional protection of threshold shifts at 48 h but not 14 days. However, IHC and OHC loss 14 days post-exposure was reduced from 30 to 46%, respectively, to 8 and 16% (Sendowski et al., 2006). Single rescue IT injections after exposure have been shown to provide functional protection of ABR thresholds and DPOAE amplitudes with accompanying HC protection when initiated within 24 h after the noise (Zhou et al., 2009; Han et al., 2015). In a patient population presenting for treatment 3 days to 2 weeks after noise exposure, IT MP injections given in conjunction with standard systemic therapy showed a larger improvement in thresholds than systemic therapy alone (Zhou et al., 2013).

Antioxidants and ROS scavengers have undergone significant investigation through multiple administration routes; however, RWM application has received less attention in part due to limited functional protection. Laurell et al. (2002) studied kinetics and distribution of radioactive D-methionine and thiourea, small sulfur-containing molecules, in rats. Both D-methionine and thiourea showed peak concentrations at the earliest sample time of 17 min with a terminal half-life of 0.57 and 0.77 h after microinfusion to the RW niche for 1 h. Tracer expression was highest in the basal stria vascularis and organ of Corti, which was attributed to the diffusion gradient, as sampling did not allow direct measurement of perilymph concentration gradients (Laurell et al., 2002). Local D-methionine solution administered to the RW niche 90 min prior to noise exposure showed a reduction in TTS at 24 h post-noise exposure. D-methionine was still present in perilymph at 24 h post-application, which was the latest time point measured (Alagic et al., 2011).

While NAC has been seen to provide significant functional and morphologic protection to noise trauma when used through other routes of administration (Kopke et al., 2007), local application has been shown to cause a temporary increase in thresholds in the basal turn (Zou et al., 2003; Eastwood et al., 2010). As such, NAC failed to provide functional or morphologic protection in guinea pigs against a vibration-induced hearing loss model (Zou et al., 2003). Bielefeld (2013) delivered an NADPH oxidase inhibitor across the RWM in chinchillas immediately prior to octave band or impulse noise. A reduction in PTS after exposure to the impulse noise was seen in treated animals; however, a significant effect was not seen for exposure to the octave band noise or in OHC counts for either exposure.

Substantial research supports that the JNK pathway underlies sensory cell death and cochlear inflammation (Eshraghi et al., 2013). D-JNK-1, a chemically synthesized cell permeable JNK ligand, has demonstrated protective effects via RWM local application when administered prior to noise exposure (Wang et al., 2003, 2007; Eshraghi et al., 2018). There is decreasing efficiency in rescue therapy in a time-dependent manner, with some efficacy of treatment seen as late as 6 h post-insult (Wang et al., 2007). While Eshraghi et al. (2018) report a “relatively flat” perilymph gradient on D-JNK-1 within hours after RWM application in the chinchilla, Wang et al. (2007) demonstrated a significant base-to-apex gradient in fluorescent labeling after one 30-min perfusion of FITC-conjugated D-JNK-1 peptides onto the RWM. In contrast to other small molecules, clearance of D-JNK-1 is relatively slow. Fluorescent staining was seen in hair cells and neurons throughout the scala tympani, with the exception of the stria vascularis, and extended into the scala vestibuli. Labeling was stable until 7 days after application, with decline starting at 14 days and limited detection at 21 days (Wang et al., 2007). However, whether strength of fluorescent staining correlates with pharmacologic activity has yet to be determined. Additionally, there is a relatively short time window for intervention blocking apoptotic processes using local application of D-JNK-1 after injury (Wang et al., 2007). Thus, extended cochlear presence may not provide additional therapeutic benefit. Prolonged exposure through intracochlear perfusion of D-JNK-1 initiated 2 days prior to exposure and continuing for 5 days after almost completely prevented permanent hearing loss and cochlear hair cell death (Wang et al., 2003). A similar effect was seen with application onto the intact RWM using either an osmotic pump or hydrogel formulation in a dose- and time-dependent manner (Wang et al., 2003; Coleman et al., 2007b). Single dose IT injections of AM-111, administered to 11 patients presenting within 24 h after firecracker injury, showed a possible therapeutic effect for some patients. However, due to the small number of patients and lack of control group, a potential effect was limited (Doz et al., 2007).

Riluzole, discussed above in systemic delivery, was also delivered intracochlearly for 2 days prior and 5 days after a 30-min noise exposure. The intracochlear riluzole treatment reduced compound action potential threshold shift and HC loss in guinea pigs. Reductions in mitochondrial damage, translocation of cytochrome c, and DNA fragmentation were also seen (Wang J. et al., 2002).

Caroverine, an AMPA and NMDA antagonist and antioxidant, has been shown to provide significant dose-dependent protection against noise in albino guinea pigs with local administration either immediately prior or up to 1 h post-exposure through blocking excitotoxic pathways (Chen et al., 2003b, 2004). However, the therapeutic window is relatively narrow, and application at 24 h failed to provide functional protection (Chen et al., 2004). Pharmacokinetics in the guinea pig after local gelfoam application of 15 μL of 1.6 or 12.8 mg/ml of caroverine showed peak perilymph concentrations 30 min post-administration, and they were 20 and 80 times higher than systemic administration of 4 mg/kg. Elimination from the perilymph was seen by 3 h for the lower dose and 6 h for the higher dose. Additionally, caroverine has been shown to cause a temporary increase in ABR thresholds and delay in ABR wave I latency up to 24 h after application, more so in the basal region with the highest assumed concentrations and at time points with highest detected perilymph concentrations, presumably through blocking glutamatergic activity (Chen et al., 2003a).

While RWM application has significant benefits due to the reduction in systemic side effects, generalizability of studies between species is limited by anatomic variation in the cochlea. Dimensions of fluid spaces vary within species (Erixon et al., 2009; Avci et al., 2014) and considerably across species (Thorne et al., 1999). Additionally, the fluid space and scala tympani is significantly longer in larger mammals, including humans, which may further limit local application of an agent to more limited regions than seen in small animal models (Wang et al., 2011; Glueckert et al., 2018). Dexamethasone perilymph concentrations at the basal turn of the cochlea were 17- to 27- fold higher in guinea pigs than sheep after 2% dexamethasone application of 50 or 600 μL, respectively, to the round window niche (Wang et al., 2011). Additionally, there is substantial inter-individual variability in response to treatment, which may be related to the high variability in perilymph concentrations measured (Bird et al., 2007).

## Conclusion

Noise exerts a complex and highly variable set of effects on the cochlea. Among those effects are those that impair the cochlea’s sensitivity to acoustic stimuli (hair cell injury, reduction of the endocochlear potential, de-afferentation of the IHCs) and those that affect the homeostasis of the organ as a whole (blood flow changes, increased permeability of the BLB). Changes in permeability of the BLB can have injurious effects, such as increasing the penetration of ototoxic compounds into scala media. However, BLB permeability changes can also have beneficial effects, such as creating a window in which drugs delivered systemically can more easily access the organ of Corti. Indeed a window for rescue interventions, in which drugs are delivered after noise to promote recovery and decrease permanent injury, are clearly effective through 12–24 h after noise. This indicates that, clinically, unanticipated noise exposures can potentially be treated to minimize hearing loss. Further, local administration of drugs can increase cochlear access to compounds that are restricted by the BLB with the goal of rendering the ear less susceptible to permanent NIHL.

## Author Contributions

All authors listed have made a substantial, direct and intellectual contribution to the work, and approved it for publication.

## Conflict of Interest Statement

The authors declare that the research was conducted in the absence of any commercial or financial relationships that could be construed as a potential conflict of interest.

## Abbreviations

BLB: blood-labyrinth barrier
CSF: cerebrospinal fluid
IHC: inner hair cell
i.p.: intra-peritoneal
IT: intratympanic
IV: intravenous
JNK: c-jun-N-terminal protein kinase
MP: methylprednisolone
NAC: n-acetyl, l-cysteine
NIHL: noise-induced hearing loss
OHC: outer hair cell
PTS: permanent threshold shift
RWM: round window membrane
s.c.: sub-cutaneous
SGN: spiral ganglion neuron
TAAC: triamcinolone acetonide
TTS: temporary threshold shift.
